# Wheat (*Triticum aestivum*) adaptability evaluation in a semi-arid region of Central Morocco using APSIM model

**DOI:** 10.1038/s41598-021-02668-3

**Published:** 2021-11-30

**Authors:** Hamza Briak, Fassil Kebede

**Affiliations:** Center of Excellence for Soil and Fertilizer Research in Africa (CESFRA), AgroBioSciences (AgBS), Mohammed VI Polytechnic University (UM6P), 43150 Ben Guerir, Morocco

**Keywords:** Plant sciences, Environmental impact, Climate change, Agroecology, Software, Statistics

## Abstract

In this study, we evaluated the suitability of semi-arid region of Central Morocco for wheat production using Agricultural Production Systems sIMulator (APSIM) considering weather, soil properties and crop management production factors. Model calibration was carried out using data collected from field trials. A quantitative statistics, i.e., root mean square error (RMSE), Nash–Sutcliffe efficiency (NSE), and index of agreement (d) were used in model performance evaluation. Furthermore, series of simulations were performed to simulate the future scenarios of wheat productivity based on climate projection; the optimum sowing date under water deficit condition and selection of appropriate wheat varieties. The study showed that the performance of the model was fairly accurate as judged by having RMSE = 0.13, NSE = 0.95, and d = 0.98. The realization of future climate data projection and their integration into the APSIM model allowed us to obtain future scenarios of wheat yield that vary between 0 and 2.33 t/ha throughout the study period. The simulated result confirmed that the yield obtained from plots seeded between 25 October and 25 November was higher than that of sown until 05 January. From the several varieties tested, Hartog, Sunstate, Wollaroi, Batten and Sapphire were yielded comparatively higher than the locale variety Marzak. In conclusion, APSIM-Wheat model could be used as a promising tool to identify the best management practices such as determining the sowing date and selection of crop variety based on the length of the crop cycle for adapting and mitigating climate change.

## Introduction

Wheat (*Triticum aestivum*) is one of the most important cereal crops and vital staple food worldwide^1,2^, for the reasons that it grows in both the temperate and warmer regions due to its resilience to drought and frosts. Moreover, wheat grain is nutritious and composed of starch, fiber, vitamins B and E, iron and antioxidants. Besides, it has a gluten content which is capable of forming the fully elastic dough required for baking leavened bread, and is also an essential ingredient globally in the food industry sector for making great varieties of food stuff^3–5^. Wheat yield in the 2019 cropping season were 3.55 t/ha in the world, 2.76 t/ha in Africa, 2.74 t/ha in North Africa and 1.61 t/ha in Morocco^6^. Compared to the year of 2018, wheat yield in the world increased by 3% in contrast to Morocco where the yield declined by 37%. As a result, Morocco has become among the top 10 wheat importing countries^7^. The decline of Morocco's wheat production in recent years has been attributed due to low and erratic precipitation with a frequent drought^8^, and unusually high mean temperature. In fact, since the world population is projected to be around 9.8 billion by 2050, wheat yield is expected to be increased by 60%^9^. The key to tackle these challenges is to adapt best management practices, which are helpful for optimizing wheat grain yield such as setting an optimum sowing date and using an appropriate wheat variety for the region^5,10–20^. Many studies confirm that the early and medium sowing date was beneficial for improving the soil water storage and increased the grain yield, and a reduction in yield and development of wheat when sowing is delayed after the optimum time, especially in a dry year^21–26^. But just a few studies which confirm that late sowing increases wheat yield^27^. Furthermore, the choice of the suitable cultivars for the specific environment is also one of the best management practices to increase the yield^5,12,15,20,28–30^.

Application of crop simulation models helps in elaborating the suitability of best management practices to boost agricultural productivity by integrating the interdisciplinary knowledge gained through experimentation and technological innovations in the fields of biological, physical, and chemical science relating to the agricultural production system^31–34^. They are widely used for decision making and planning in agriculture and can be particularly useful for predicting food production in response to climate change^35–37^. The Agricultural Production Systems Simulator (APSIM)^38^ is one of the most appropriate models for agricultural systems. The model has been used successfully for simulating crop adaptation, efficient and sustainable production. It is particularly designed and suited for assessing the impacts of alternative management practices on the soil properties and crop productivity through the linking of crop growth with soil processes in different climates^39,40^. APSIM is also concerned about the long-term repercussions of the actions of farmers, for example, on yield levels and soil nutrient status. Keating et al.^38^ noted that the main thrust of APSIM is a combination of crop yield estimation, as a result of how farmers manage their farming systems, and the effects of these management decisions in the long run. To establish the applicability of the APSIM model, it is necessary to evaluate it in order to assess its capability to predict experienced outcomes in the real world. APSIM model has been used to simulate the performance crop production under stress conditions over the world and in various environments such as arid, semi-arid and Mediterranean climates^41–46^. APSIM’s performance was statistically assessed against field trial data. After properly parameterizing, the model performs well in simulating the yield limiting factors. Nevertheless, APSIM model has not been tested yet in the Moroccan context, thus far. Therefore, this work was carried out to evaluate the APSIM’s applicability to simulate wheat production factors in Central Morocco.

The overall goal of this study was to evaluate wheat production in Central Morocco as a function of weather, soil properties and crop management using APSIM model.

## Materials and methods

### Study of the site description

**Figure 1:**
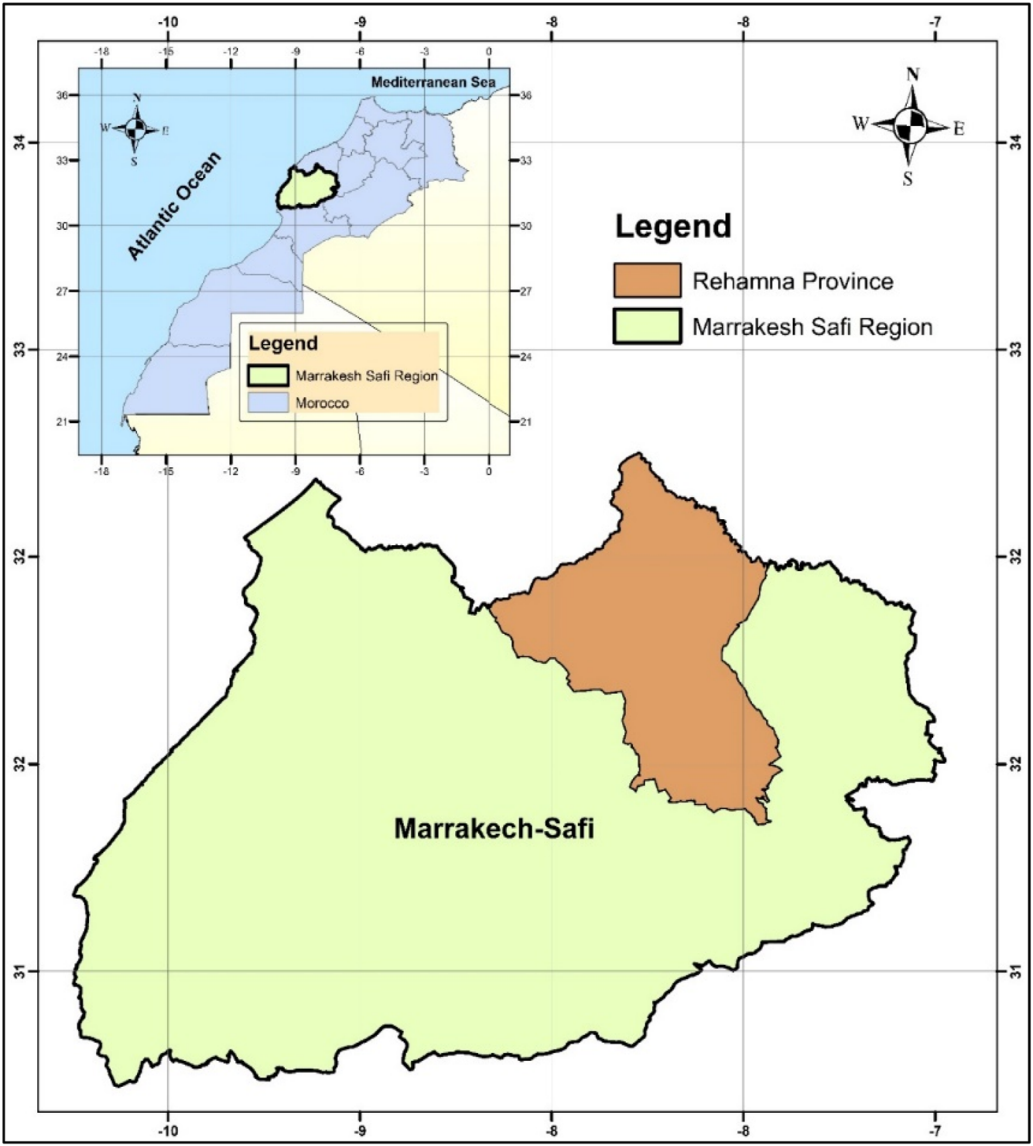
Geographical location of Rhamna region.

The study was conducted in the Marrakech Safi region, which is located in Central Morocco (Fig. 1), covering an area of 41,404 km^2^, which represents 6% of the national territory. This region consists of seven provinces (i.e., Safi, Al Haouz, El Kelâa des Sraghna, Rhamna, Youssoufia, Chichaoua and Essaouira), where 23.6% of the national farmlands are located (i.e., 5,821,800 hectares)^47^. Fundamentally, this study focuses on the Rhamna province as it is a number one cereal and legume growing belt, which accounts for 16.81% of the farmland of Morocco. In fact, it stands sixth position in term of the total regional cereal production (8.74%) and fifth in terms of the total regional legume production (4.74%)^48^. Rhamna covers 5877 km^2^ with a population of 315,077 inhabitants, and it is a semi-arid region with fertile soil and limited water resources. However, the agricultural sector, which is one of the pillars of the regional economy, faces several problems, namely: the aridity of the climate, the poor structuring of irrigation water and the salinization of agricultural land, which limits the development of modern agriculture to high yield.

The map (Fig. 1) was edited using ArcGIS software (10.6 version), based on input vector layers of Moroccan administrative units (ESRI shapefile format). The input data was downloaded from (www.geodatashp.com).

### APSIM model structure and research design

**Figure 2:**
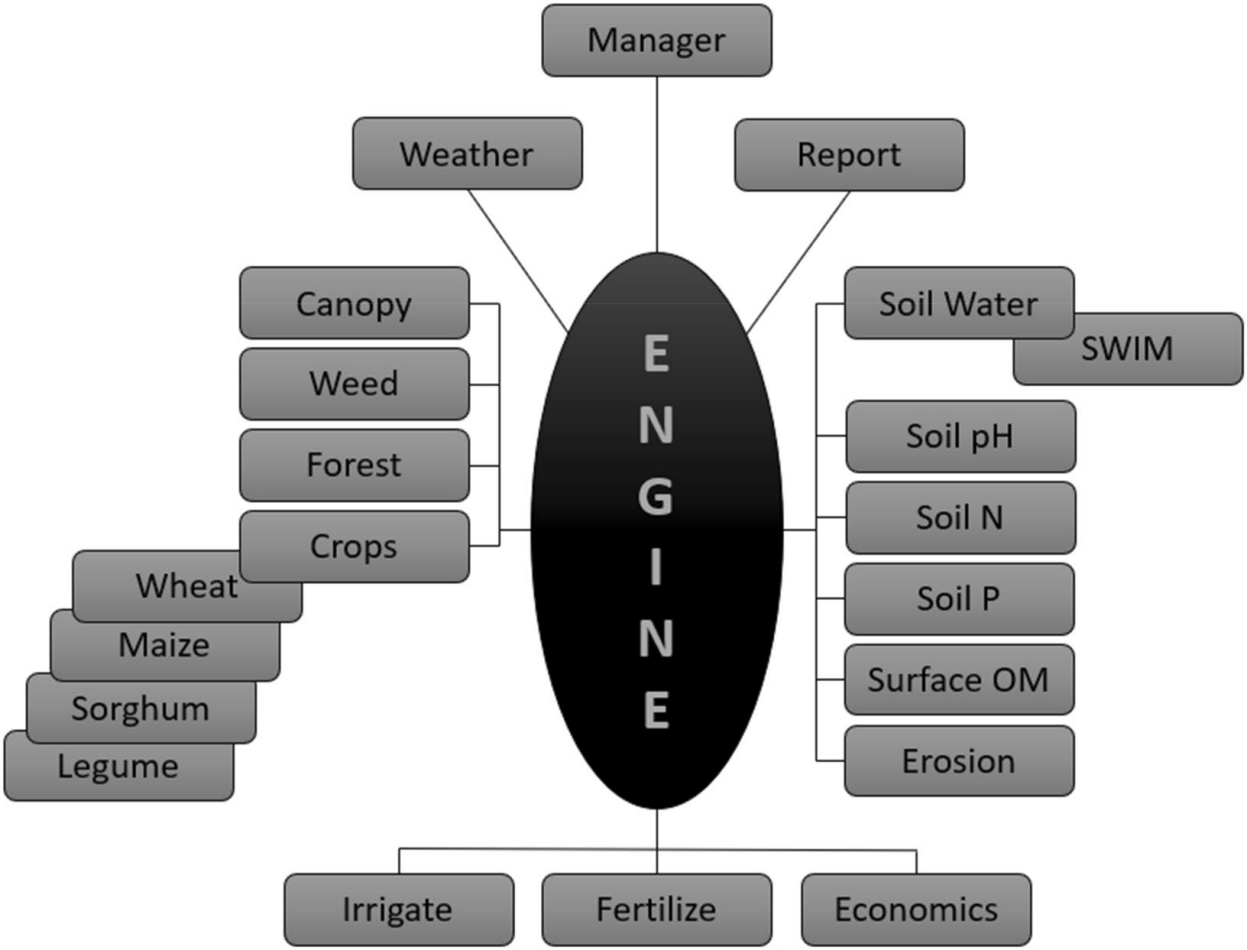
Modules and engine of APSIM^38,53,69,70^.

The **A**gricultural **P**roduction **S**ystems s**IM**ulator (**APSIM**) is a dynamic farming systems simulation model that combines biophysical and management modules within a central engine (Fig. 2) on a daily time-step^38,49–51^. According to Climate Kelpie^52^, APSIM simulates effects of environmental variables and farm management decisions on crop yield and profits. The fact that APSIM is made out of different soil modules, a range of crop modules and crop management options under different climates makes it an accurate tool for predicting crop yields, if all the data input is done correctly. This also implies that it can be used everywhere in the world, including in small-scale farming systems of Africa, as long as it is validated for local conditions and crops. The development of the APSIM model was firstly focused on the estimation of crop yield as influenced by the availability of water and nitrogen^53^, but it expanded to include other agricultural systems and environmental processes^54–56^. The suite of modules that contains the APSIM software framework enable the simulation of farming systems for a diverse range of plant^57^, crop types^58^, cropping systems rotations^59^, management^60^, soil water^61^, soil organic carbon^62^, soil nutrients^63^, animals^64^, trees^65^, climate^42,66^ and Genotype * Environment * Management interactions^67^. The simulator is recognized worldwide as a highly advanced platform for modelling and simulation of agricultural systems^68^. In this study, the APSIM model v7.10 (www.apsim.info) was used for predicting the wheat yield at different sowing dates, as well as assessing the reliability of the simulations against the measured yield data.

### Data input for APSIM

#### Observed climate data

The daily measured climatic data (rainfall and temperature) were collected from the meteorological station (32° 15′ 10.2″ N; 7° 57′ 11.9″ W) of National Office of Agricultural Council (ONCA) in Ben Guerir, while the daily radiation was estimated from solar radiation data website (www.soda-pro.com). The climate of the Rhamna region is distinguished by apparent variability.

**Figure 3:**
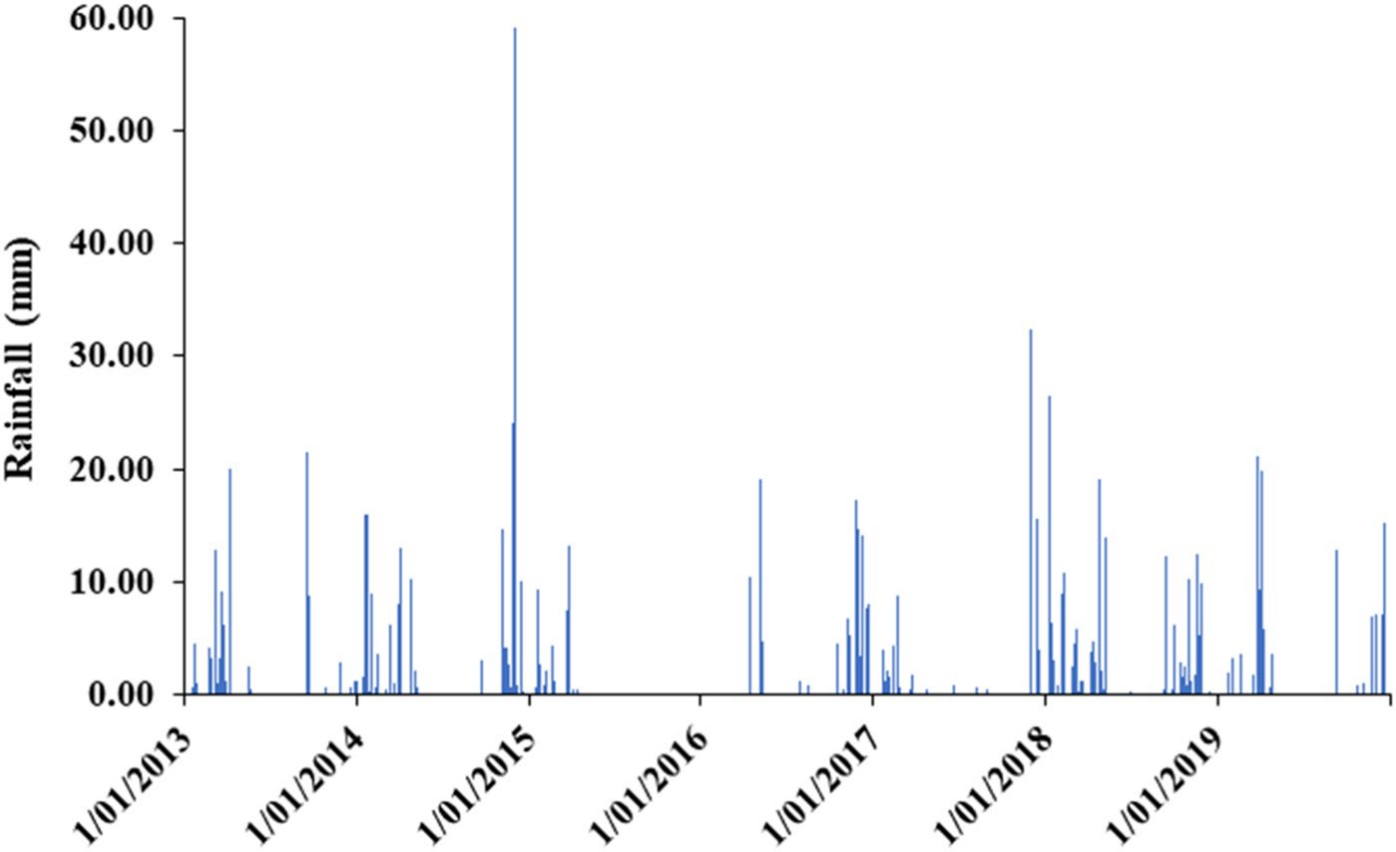
The evolution of rainfall over 7 years’ time horizon (2013–2019).

During the period of this study from 2013 to 2019 (i.e., 7 years), the mean annual rainfall of the province is 168 mm; which is low in quantity and erratic in distribution as well. The lowest precipitation is obtained in July (0.2 mm), whereas the highest is in the month of November, which is 42.24 mm (Fig. 3).

**Figure 4:**
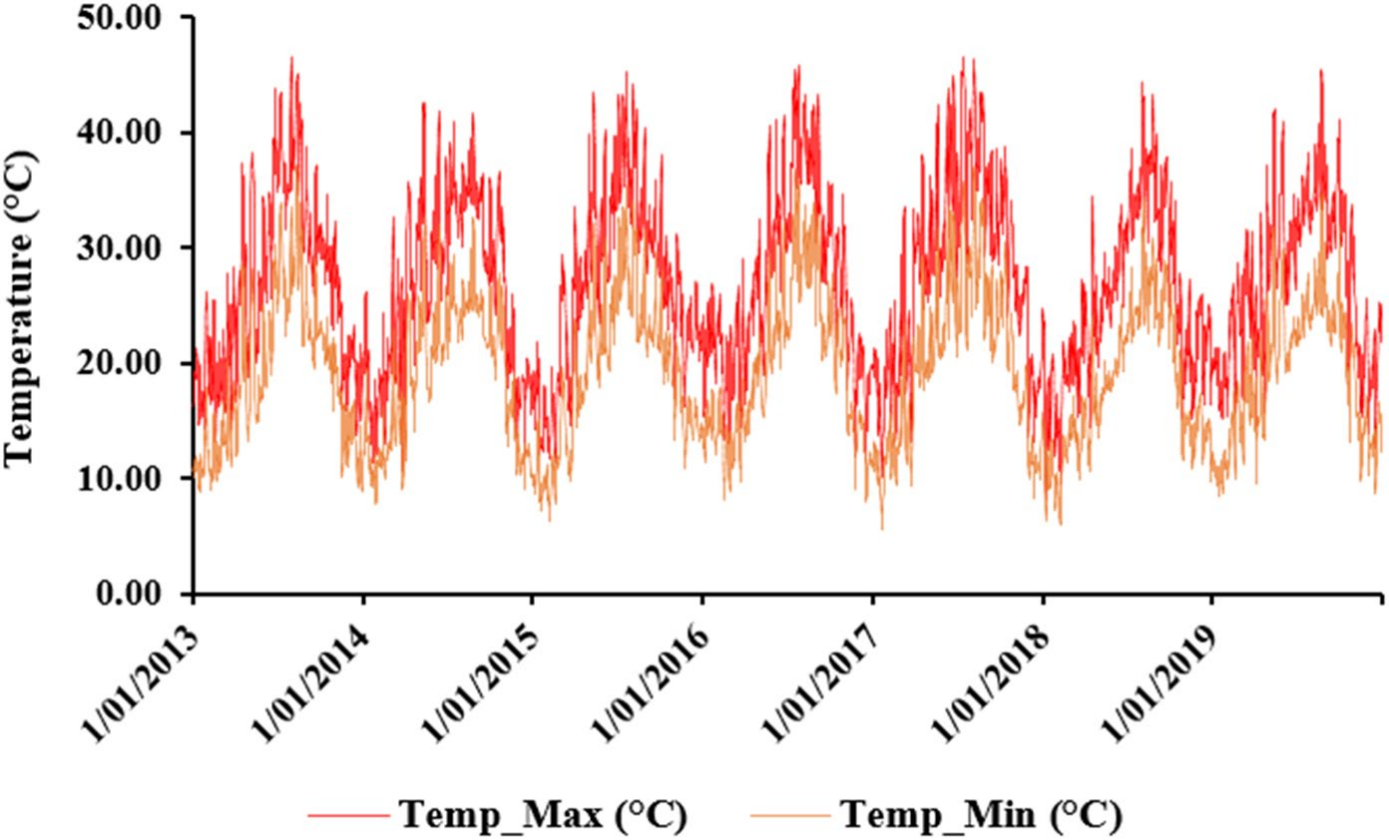
The evolution of maximum and minimum temperature over 7 years’ time horizon (2013–2019).

Besides, the annual average daily maximum and minimum temperature are 27.03 °C and 19.12 °C, respectively. August is the hottest month of the year with a maximum and minimum average daily temperature of 37.02 °C and 27.72 °C, respectively. January is therefore the coldest month of the year with a maximum and minimum average daily temperature in the range respectively of 18.14 °C and 11.37 °C (Fig. 4).

**Figure 5:**
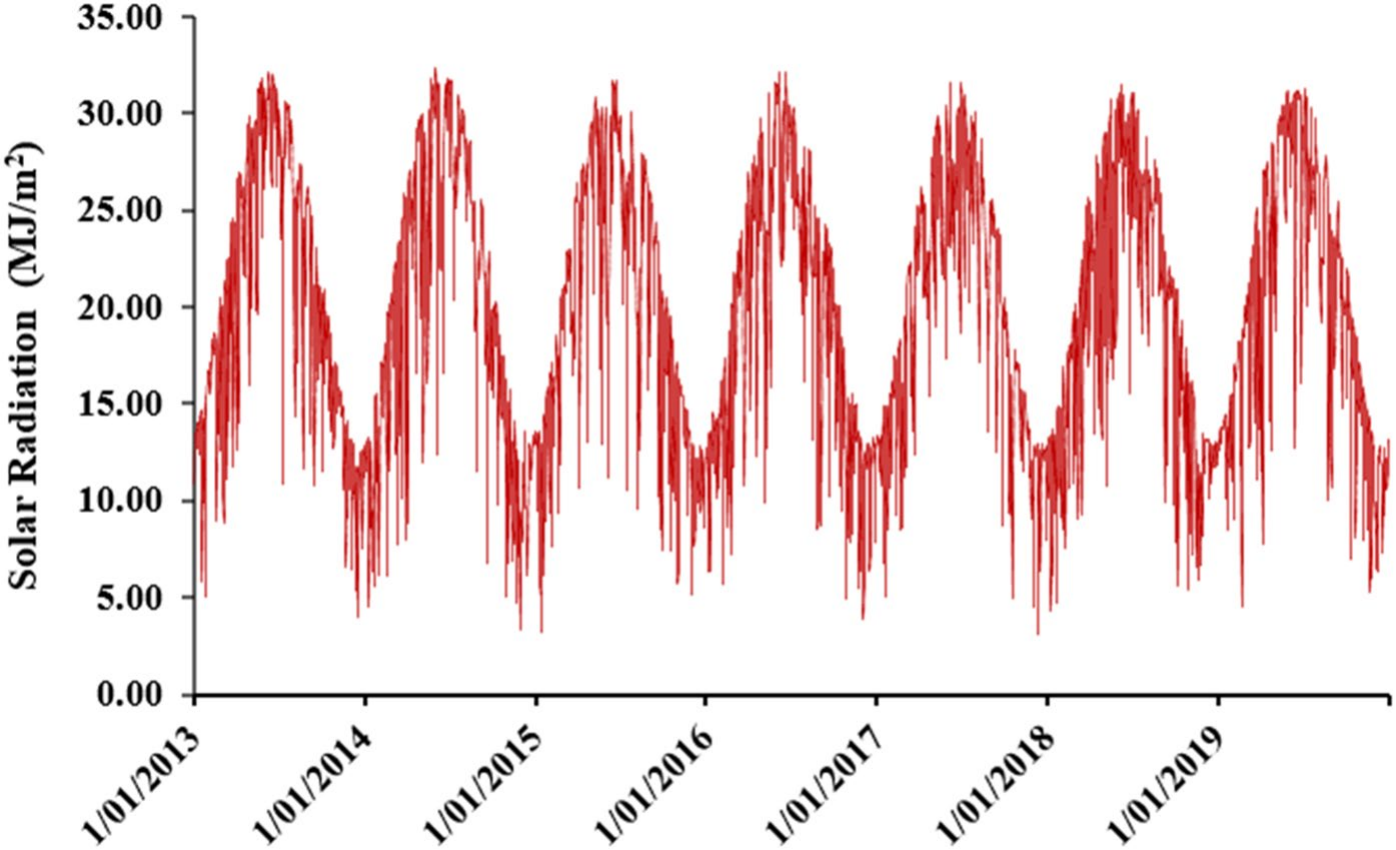
The evolution of solar radiation over 7 years’ time horizon (2013–2019).

With regard to radiation, Fig. 5 shows the daily solar radiation for 7 years. The annual average daily solar radiation during this period is 19.71 MJ/m^2^. The highest monthly averaged radiation was detected in June with a value of 28.15 MJ/m^2^, and the lowest radiation was in December with a value of 11.21 MJ/m^2^.

#### Future climate data

To study natural phenomena, such as those occurring in our environment (e.g., climate change), repeated observations of everyone in a population, ordered in time and space, are generally used. Two factors of variability appear on repeated data sets: the variability between observations measured on the same individual and the variability between the individuals themselves. The mixed model is a statistical approach that allows the highlight a relationship between the observed response and the explanatory covariates, taking into account these two types of variation^71–74^. The general mixed models that will be used in this study, are presented in (Eq. 1) according to Laird and Ware^75^ and Littell et al.^76^.

1 $${\mathbf{y}}_{{\mathbf{i}}} = \, {\mathbf{X}}_{{\mathbf{i}}} {{\varvec{\upbeta}}} \, + \, {\mathbf{Z}}_{{\mathbf{i}}} {\mathbf{U}}_{{\mathbf{i}}} + \, {{\varvec{\upvarepsilon}}}_{{\mathbf{i}}} ,$$

where for each individual i, E (y_i_ | U_i_) = X_i_β + Z_i_U_i_ is the conditional mean of y_i_ given U_i_, Z_i_ is a matrix n_i_ × q of incidence of random effects U_i_, we assume that U_i_ ~ N (0, D), where 0 is the vector of dimension q × 1, D is the covariance matrix of u. We suppose that u and ε are independent. We still have the variance of y conditionally to u is Var (y_i_ | U_i_) = Var (ε_i_) = R_i_.

Based on the graph, all the months show the same behavior over time, which reflects one of the limitations of the fixed-effects model, which assumes independence between observations. To consider the dependence and the hierarchical structure of the data, we add a random effect to the fixed-effects model, the model thus obtained is called a multilevel mixed-effects model.

- *For temperature and radiation*

2 $${\mathbf{Yijkm}} = {\mathbf{\beta 0}} + {\mathbf{\beta 1}}*{\mathbf{A}} + {\mathbf{\beta 2}}*{\mathbf{B}} + {\mathbf{\beta 3}}*{\mathbf{C}} + {\mathbf{\beta 4}}*{\mathbf{D}} + {\mathbf{b0i}} + {\mathbf{b0}}\left( {\mathbf{j}} \right){\mathbf{i}} + {\mathbf{b0}}\left( {{\mathbf{jk}}} \right){\mathbf{i}} + {\mathbf{\varepsilon ijkm}}$$

β0, β1, β2, β3 and β4 represent the fixed effects. εijkm represents the random, the index i indicates the level 1 observations, j level 2 and k those of level 3, Tijkm is then the kth observation of the temperature/radiation variable for the day (i) in the month (j) in the year (k), β0 represents the “standard” or average temperature/radiation of a day at the starting time (Zero Time). The term b0i constitutes the random effect specifying the day i associated with the intercept and representing the variations in the temperature/radiation of the day with respect to the start time. The term b0(j)i constitutes the random effect specifying the month j including the day i associated with the intercept. The term b0(jk)i constitutes the random effect specifying the year k and month j including the day i associated with the intercept.

A = Time, B = Maxt, C = Mint & D = rainfall.

- *For precipitation*

3 $${\mathbf{Tijk}} = {\mathbf{\beta 0}} + {\mathbf{\beta 1}}*{\mathbf{cos}}*\left( {{\mathbf{wXijk}}{-}{\mathbf{\phi T}}} \right) + {\mathbf{b0i}} + {\mathbf{b0}}\left( {\mathbf{j}} \right){\mathbf{i}} + {\mathbf{\varepsilon ijk}}$$

With: w = 0.2, ϕT = 20, T and X are fixed, β0, β1 represent fixed effects. The parameters β0, β1 and σε are estimated by the least squares method.

The subscripts i and j denote respectively the level 1 and 2 observations, Tijk is then the kth observation of the precipitation variable for month (i) in year (j), β0 corresponds to the "standard" or average precipitation of a month at the starting time (time zero). The term b0i represents the random effect specifying month (i) associated with the β0 intercept representing the months precipitation variations from the starting time. The term b0(j)i is the random effect specifying year j including month i associated with the intercept β0. β1 also represents the average increase in precipitation per unit time.

#### Soil data

A survey was carried out to collect both disturbed and undisturbed soil samples from the surface and subsurface horizons from wheat fields in the Rhamna province. Moreover, field measurement such as soil color determination was carried out by using a Munsell chart. Furthermore, in accordance with the soil data requirement for APSIM model, the physico-chemical soil properties were determined such as texture using a hydrometer according to the Bouyoucos^77^ method; bulk density (BD) by the core method; soil moisture content by the gravimetric method; pH-water by the potentiometric method; electrical conductivity (EC) using the EC meter in a 1:5 soil to water ratio; organic carbon (OC) by the dry combustion; nitrate (NO_3_^−^) & ammonium (NH_4_^+^) by the scalar method and cation exchange capacity (CEC) by the cobaltihexamine method.

The study revealed that the soils of Rhamna are dominantly sandy clay loam, reddish brown in color, highly compacted soil due to a higher bulk density, alkaline pH, non-saline, low in carbon, moderate in nitrogen and CEC (Table 1).

**Table 1:** Soil physical and chemical properties of the Rhamna region.

Depth (cm)	Clay (%)	Silt (%)	Sand (%)	Texture	Munsell Color	BD (g/cm^3^)	SMC (%)	pH (1:5 water)	EC (1:5 dS/m)	OC (%)	NO_3_^−^ (kg/ha)	NH_4_^+^ (kg/ha)	CEC (cmol + /kg)
0–20	20	22	58	Sandy Clay Loam	Reddish Brown (5YR 4/4)	1.45	3.00	8.51	0.54	0.77	14.29	2.35	17.90
20–50	18	20	62	Sandy Loam	Reddish Brown (5YR 4/4)	1.46	3.01	8.53	0.54	0.77	53.15	8.74	18.20

### Evaluation of the APSIM Model

Linear regression was used to compare graphically and to analyze statistically measured and predicted data of wheat yield, in order to evaluate the adaptability and the performance of the APSIM model. The statistical indices which are the coefficient of determination (**R**^2^), the root mean square error (**RMSE**) [Eq. 4], the Nash–Sutcliffe efficiency (**NSE**)^78^ [Eq. 5] and the index of agreement (**d**)^79^ [Eq. 6], are defined as follows:

4 $${\mathbf{RMSE}} = \left[ {\mathop \sum \limits_{{{\text{i}} = 1}}^{{\text{n}}} \frac{{\left( {{\text{Y}}_{{\text{i}}}^{{{\text{obs}}}} - {\text{ Y}}_{{\text{i}}}^{{{\text{sim}}}} } \right)^{2} }}{{\text{n}}}} \right]^{0.5}$$

5 $${\mathbf{NSE}} = 1 - { }\frac{{\mathop \sum \nolimits_{{{\text{i}} = 1}}^{{\text{n}}} \left( {{\text{Y}}_{{\text{i}}}^{{{\text{obs}}}} - {\text{ Y}}_{{\text{i}}}^{{{\text{sim}}}} } \right)^{2} }}{{\mathop \sum \nolimits_{{{\text{i}} = 1}}^{{\text{n}}} \left( {{\text{Y}}_{{\text{i}}}^{{{\text{obs}}}} - {\text{ Y}}^{{{\text{mean}}}} } \right)^{2} }}$$

6 $${\mathbf{d}} = 1 - { }\frac{{\mathop \sum \nolimits_{{{\text{i}} = 1}}^{{\text{n}}} \left( {{\text{Y}}_{{\text{i}}}^{{{\text{obs}}}} - {\text{ Y}}_{{\text{i}}}^{{{\text{sim}}}} } \right)^{2} }}{{\mathop \sum \nolimits_{{{\text{i}} = 1}}^{{\text{n}}} \left( {\left| {{\text{Y}}_{{\text{i}}}^{{{\text{sim}}}} - {\text{ Y}}^{{{\text{mean}}}} } \right|{ } + { }\left| {{\text{Y}}_{{\text{i}}}^{{{\text{obs}}}} - {\text{ Y}}^{{{\text{mean}}}} } \right|} \right)^{2} }}$$

where ***Yi ***^***sim***^ is the predicted value, ***Yi ***^***obs***^ is the observed value, ***Y***^***mean***^ is the mean of the observed values and ***n*** is the number of observations.

For good model performance, values of **RMSE** [Eq. 4] should be close to 0, that indicate the better agreement between the two variables^80^. The agreement value of 1 indicates a perfect match of the simulated to the observed data for **NSE** [Eq. 5]^78^ and **d** [Eq. 6]^81^. Vice versa, 0 indicates no agreement for both.

### Model validation

Various simulations for wheat yield were conducted by APSIM model Version 7.10 under natural rainfed conditions for Marzak cultivar, which is the common variety mostly used in the Rhamna region—Central Morocco. They were carried out using: (i) a daily weather data for 7 years (2013 to 2019), (ii) soil data, (iii) water balance parameters calculated and/or estimated as described by/in previous studies^82–90^, and (iv) growth and management crop data retrieved from the field survey. In fact, a calibration of the APSIM model was realized until obtaining a simulated wheat yield that fully matches with the measured wheat yield of Rhamna region, which were collected from the Regional Office for Agricultural Development of Haouz, Morocco (ORMVAH). Indeed, the projected climate data (2020 to 2030) which is realized by the statistical mixed model was integrated in the calibrated APSIM model to obtain the future scenarios of wheat yield. Therefore, different sowing dates were simulated using the calibrated APSIM model to analyze their effects on wheat yield. Wheat may be sown early due to the limitation of the available water, and it may be sown at a medium or late date due to delay in harvesting previous crops and/or rainfall. However, according to the regional sowing window, the sowing dates started on 25 October and were repeated every 10 days until 5 January. Moreover, several simulations with various varieties of wheat under the same conditions were analyzed to choose the most suitable cultivar for the region with high yield.

## Results

### Climate change scenarios

#### Adjustment of the model (2)

**Figure 6:**
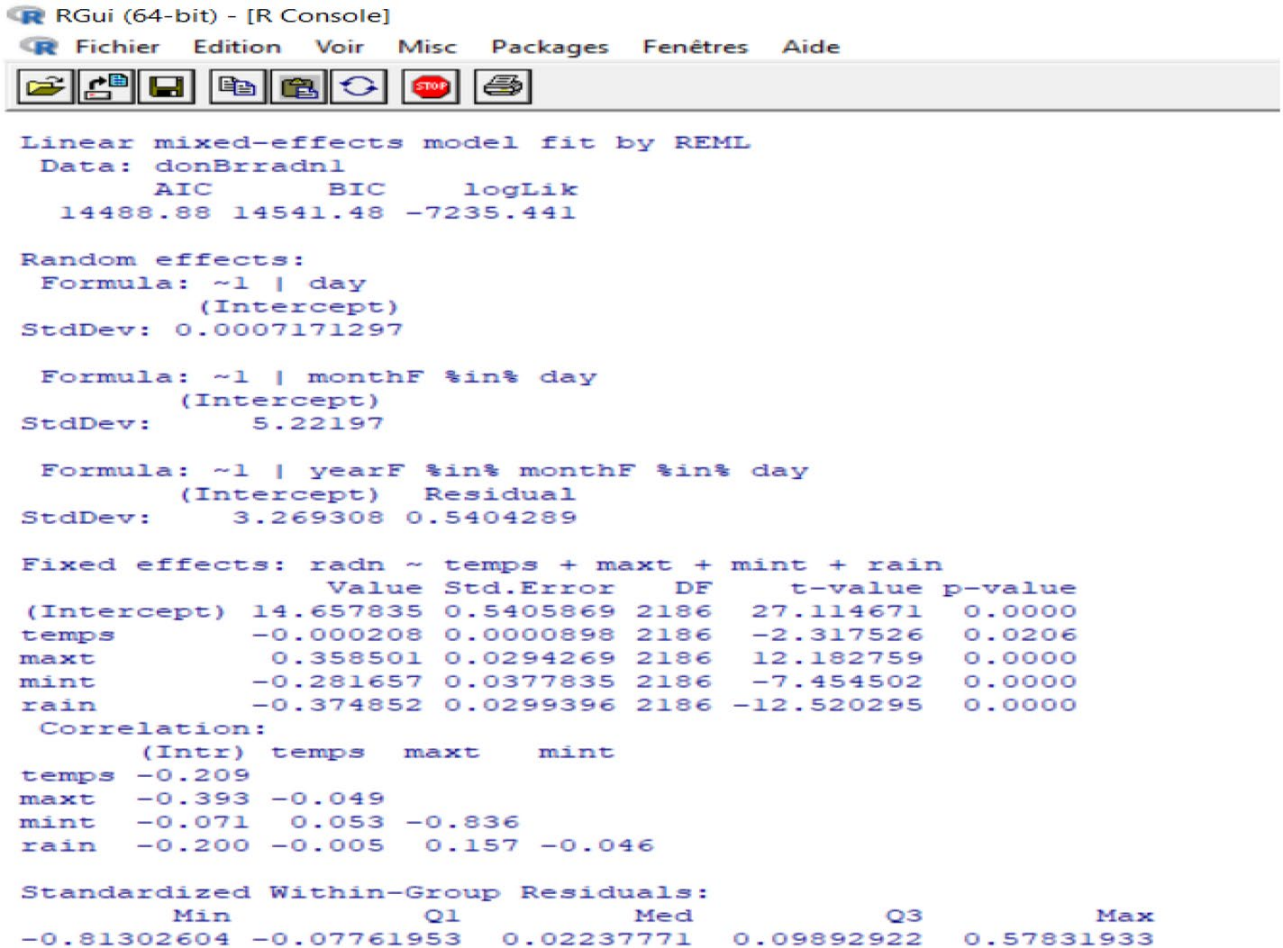
Summary of the model (2).

The fitting of the second model gives the following results (Fig. 6):

According to the synthesis results, the p-value is lower than 0.05 for all parameters of the fixed effect of radiation, temperature and rain. Therefore, the obtained values for the random effect and error parameters agree.

**Figure 7:**
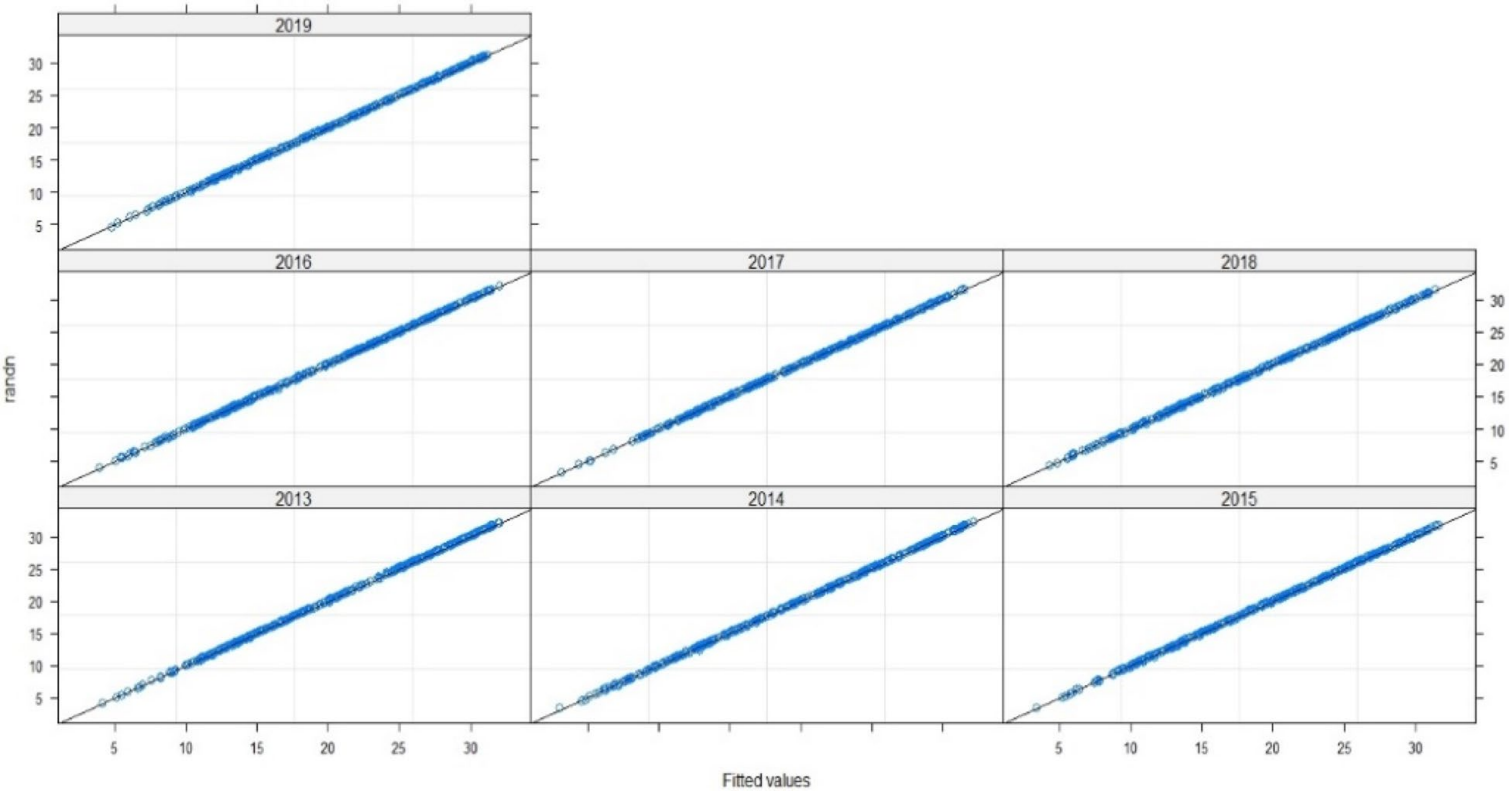
Radiation in terms of the adjusted values.

**Figure 8:**
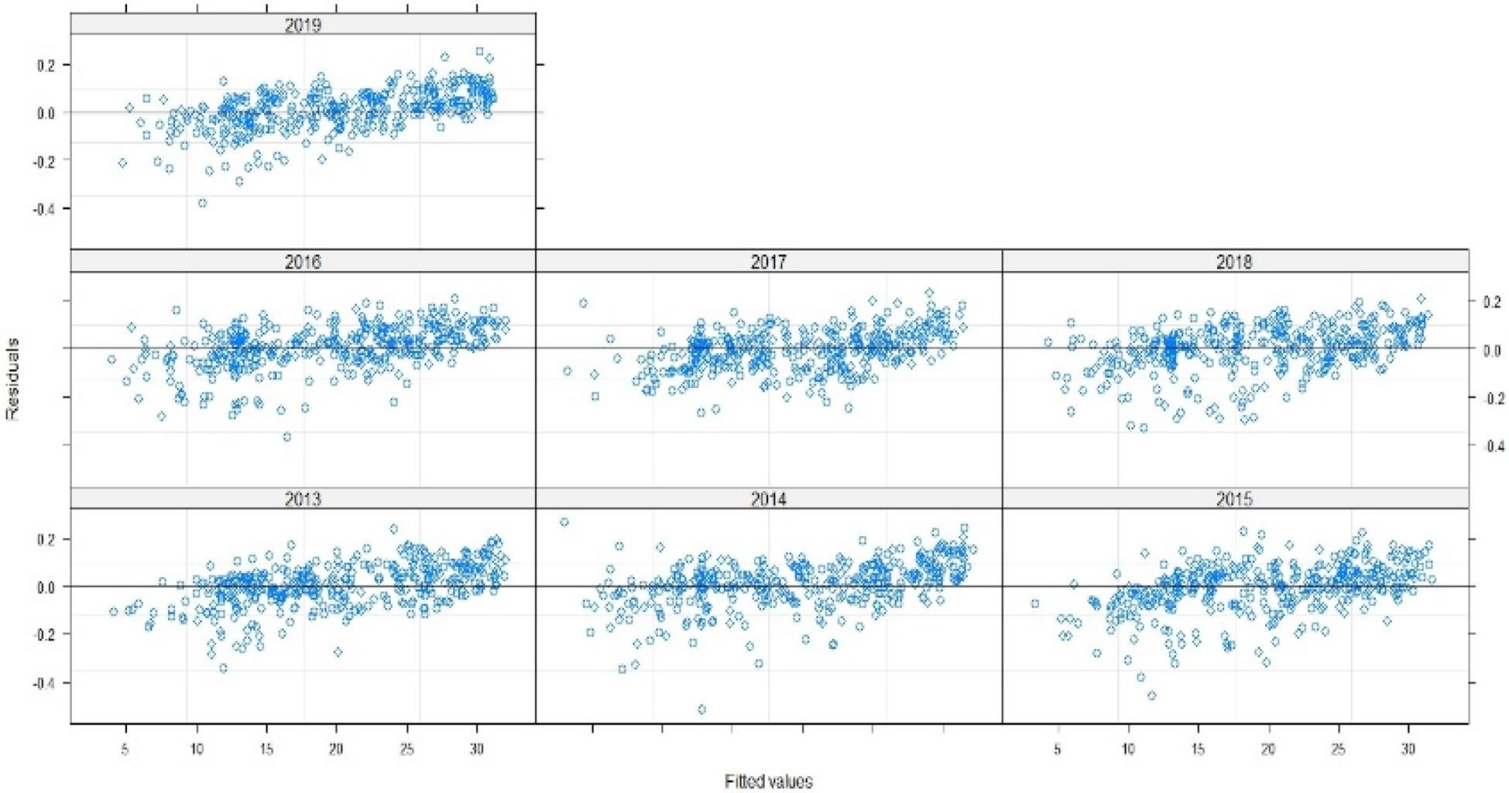
Errors in terms of the adjusted values.

**Figure 9:**
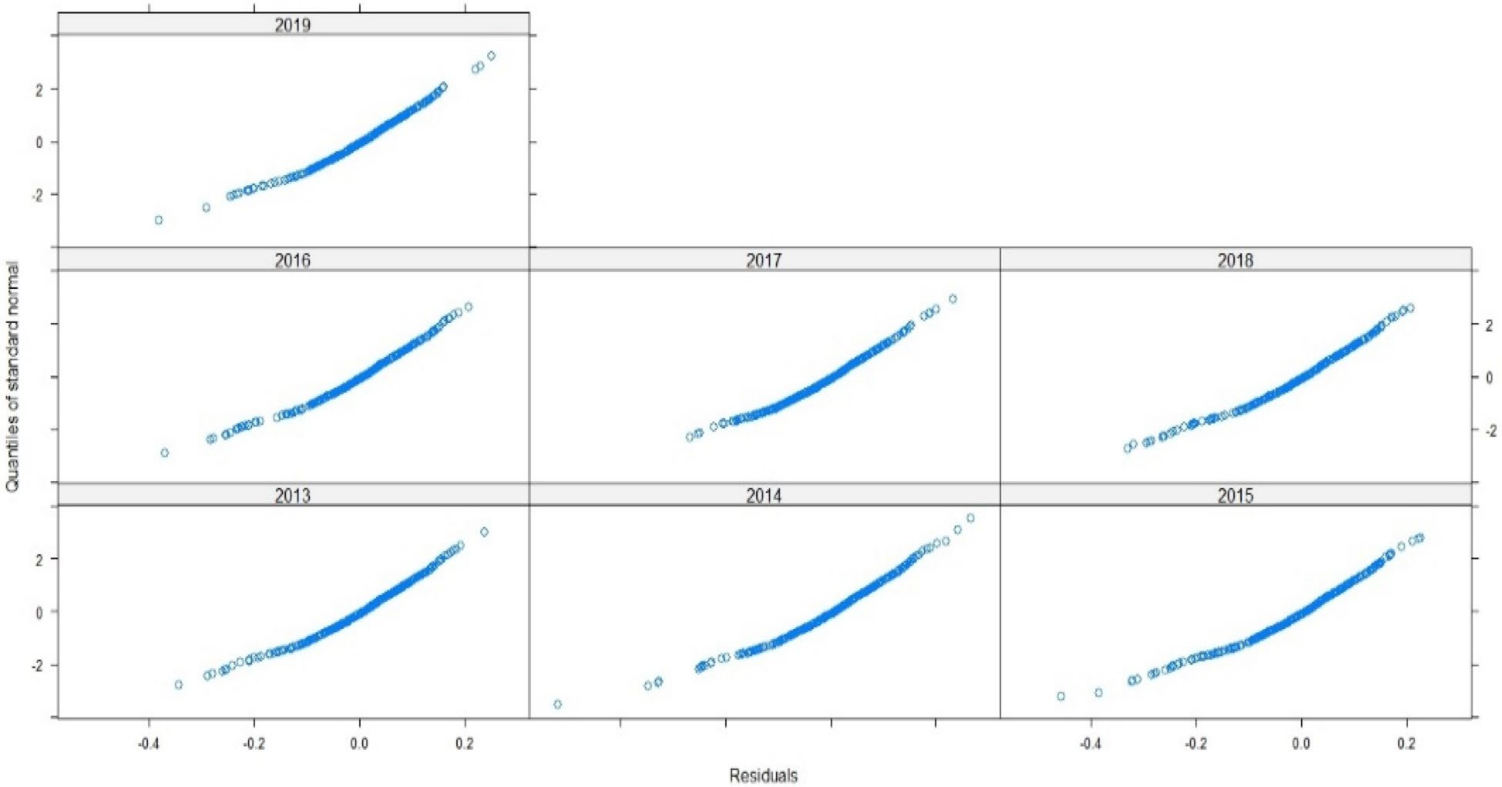
The standard Normal.

Thus, to assert the validation of the model, we need to examine the fit of the fitted values and errors, and to check the normal distribution line: The points are well fitted by contribution to normality, and the samples follow the normal distribution by perfection. Thus, the measurement errors are also aligned in terms of mean (Figs. 7, 8, 9). Therefore, the multilevel linear mixed effect model is suitable for the sample we want to treat.

#### Adjustment of the model (3).

**Figure 10:**
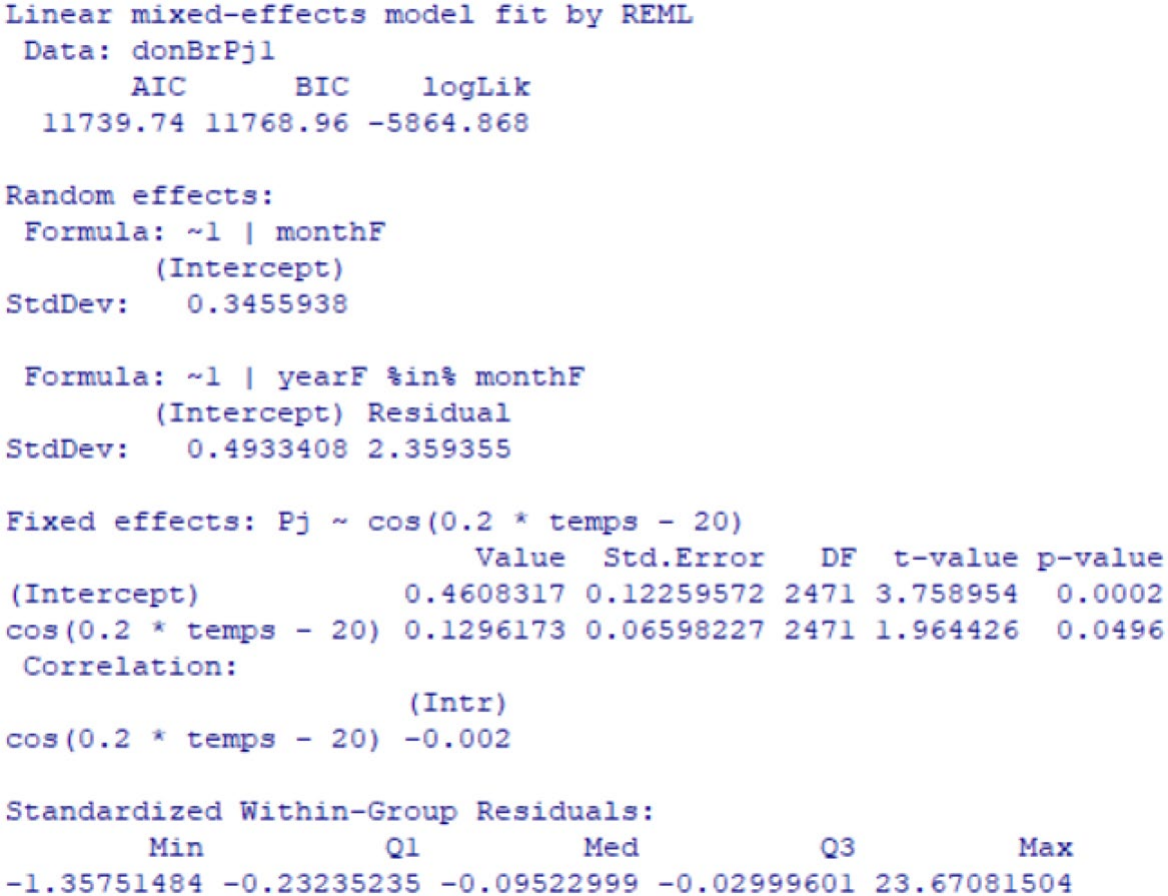
Summary of the model (3).

Consistent with the simulation results, the p-value is also less than 0.05 for all precipitation fixed effect parameters (Fig. 10). Furthermore, the values obtained for the random effect and error parameters are consistent.

**Figure 11:**
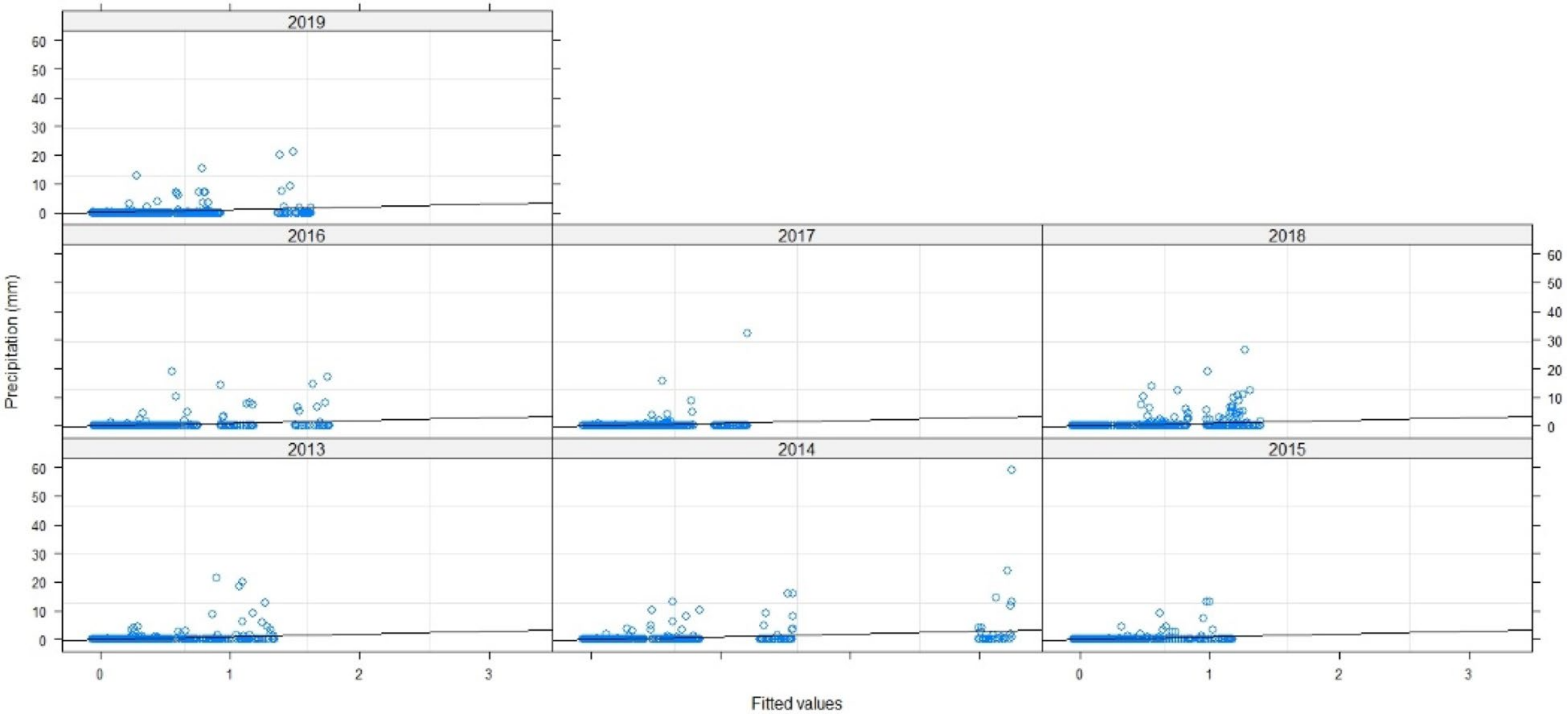
Precipitation in terms of the adjusted values.

**Figure 12:**
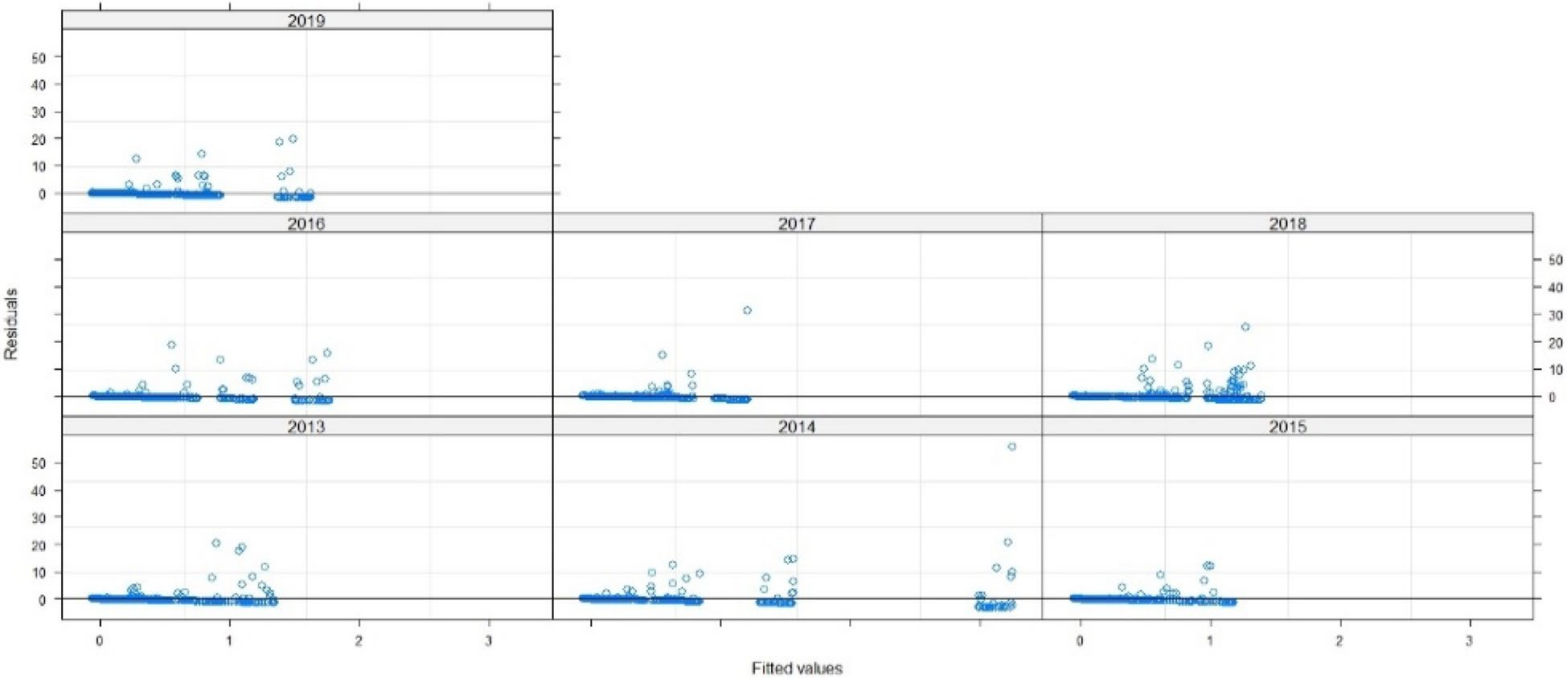
Errors in terms of the adjusted values.

Furthermore, to affirm the validation of the model, we need to examine the fit of the fitted values and errors, and check the normal distribution line: The points are well fitted with respect to normality. Thus, the measurement errors are also aligned in terms of mean (Figs. 11, 12). Therefore, the multilevel linear mixed effect model is suitable for the sample we want to treat.

#### Future scenarios

**Figure 13:**
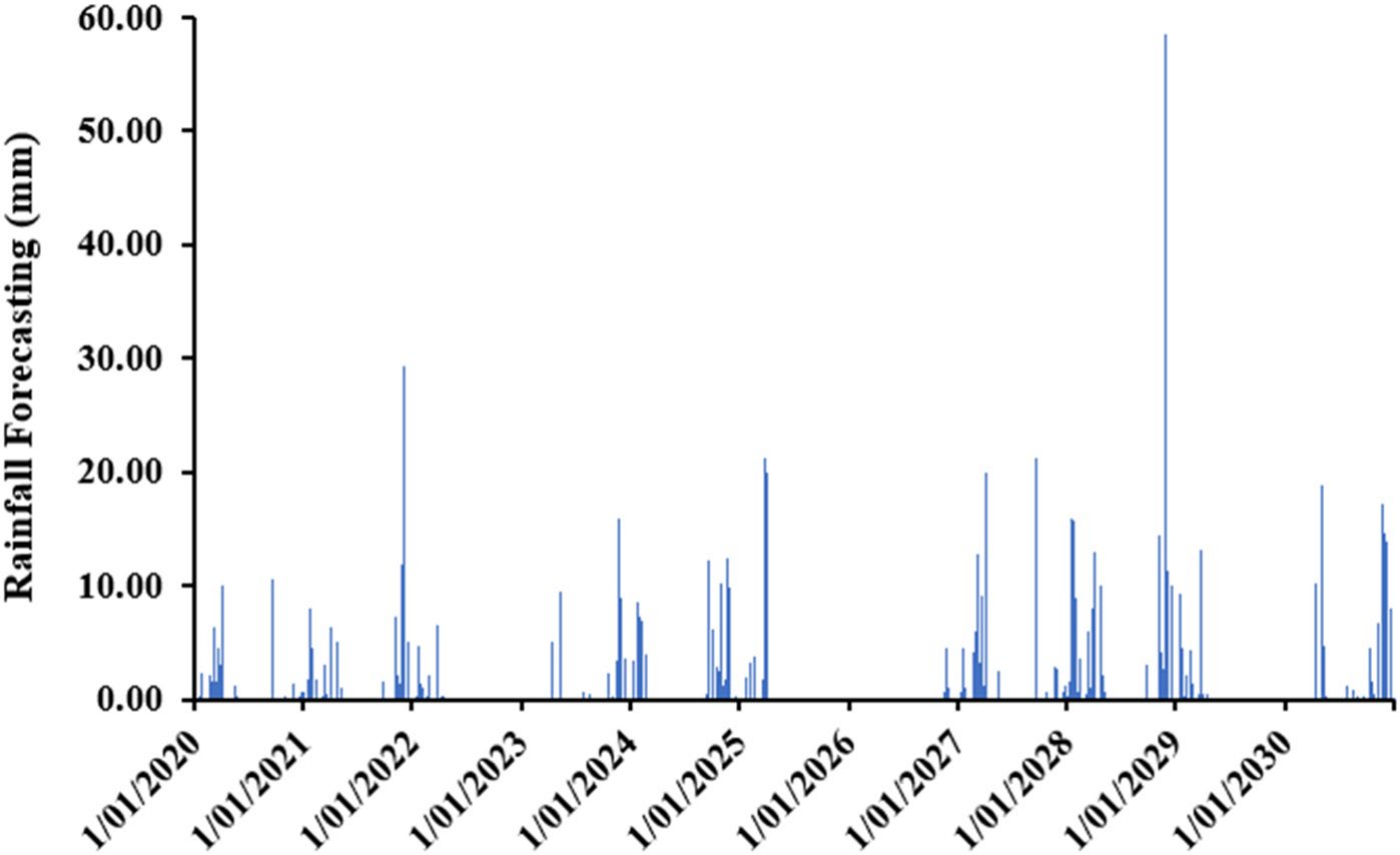
The evolution of rainfall over future years (2020–2030).

The results of future scenarios for the Rhamna region from 2020 to 2030 showed that the mean annual rainfall is about 113 mm, which is low in quantity and erratic in distribution as well. The lowest precipitation is obtained in June (0.05 mm), whereas the highest is in the month of November, which is 35.84 mm (Fig. 13).

**Figure 14:**
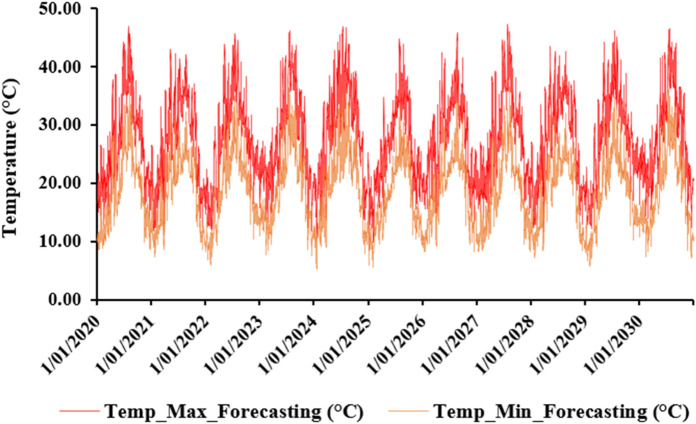
The evolution of maximum and minimum temperature over future years (2020–2030).

The annual average daily maximum and minimum temperature are 27.61 °C and 18.67 °C, respectively. August is the hottest month of the year, with a maximum and minimum average daily temperature of 37.50 °C and 27.20 °C, respectively. January is therefore the coldest month of the year with a maximum and minimum average daily temperature in the range respectively of 18.86 °C and 11.11 °C (Fig. 14).

**Figure 15:**
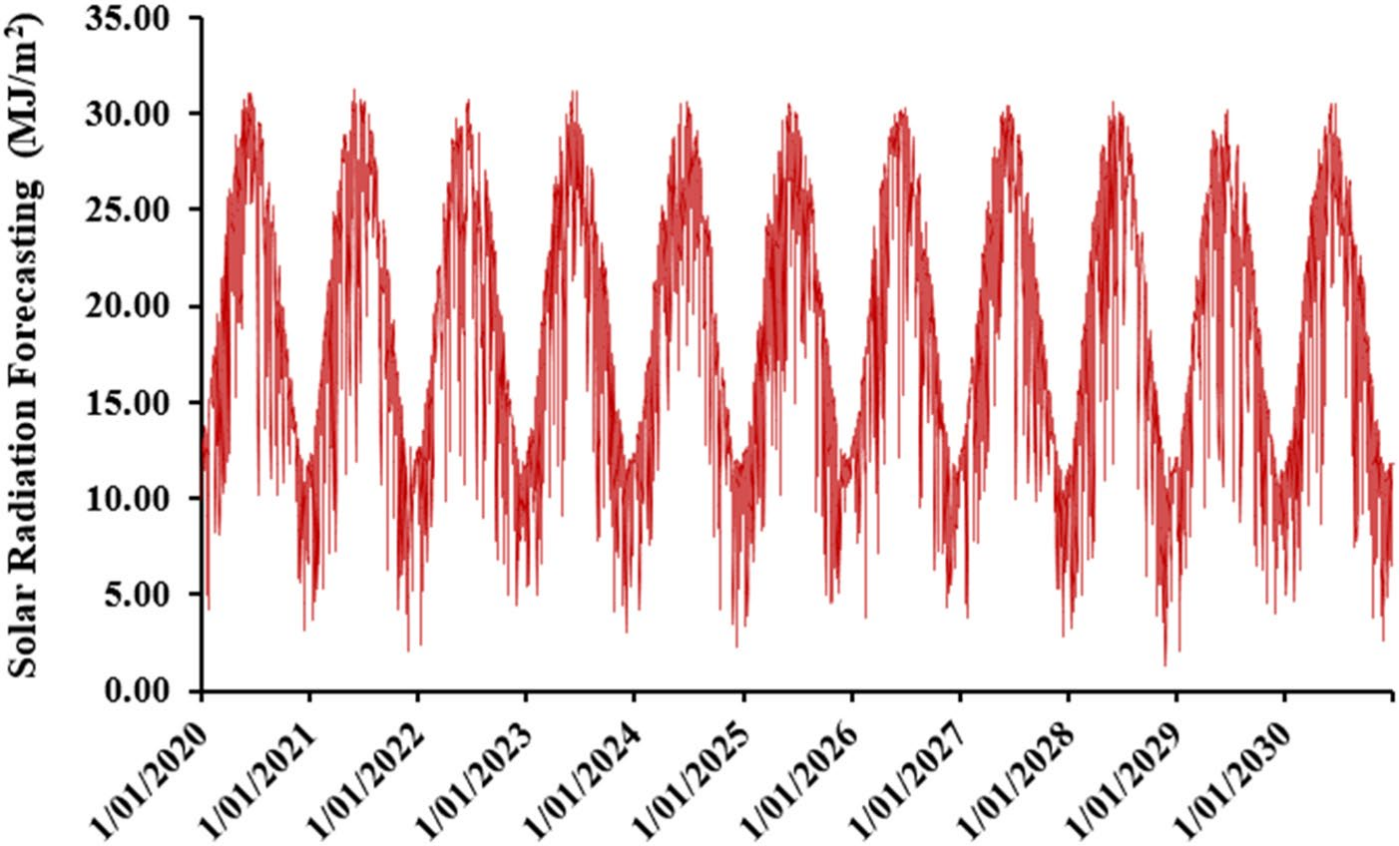
The evolution of solar radiation over future years (2020–2030).

Figure 15 shows the daily solar radiation for the future years (2020–2030). The annual average daily solar radiation during this period is 18.61 MJ/m^2^. The highest monthly averaged radiation was detected in June with a value of 27.06 MJ/m^2^, and the lowest radiation was in December with a value of 10.09 MJ/m^2^.

### Performance of APSIM model

**Figure 16:**
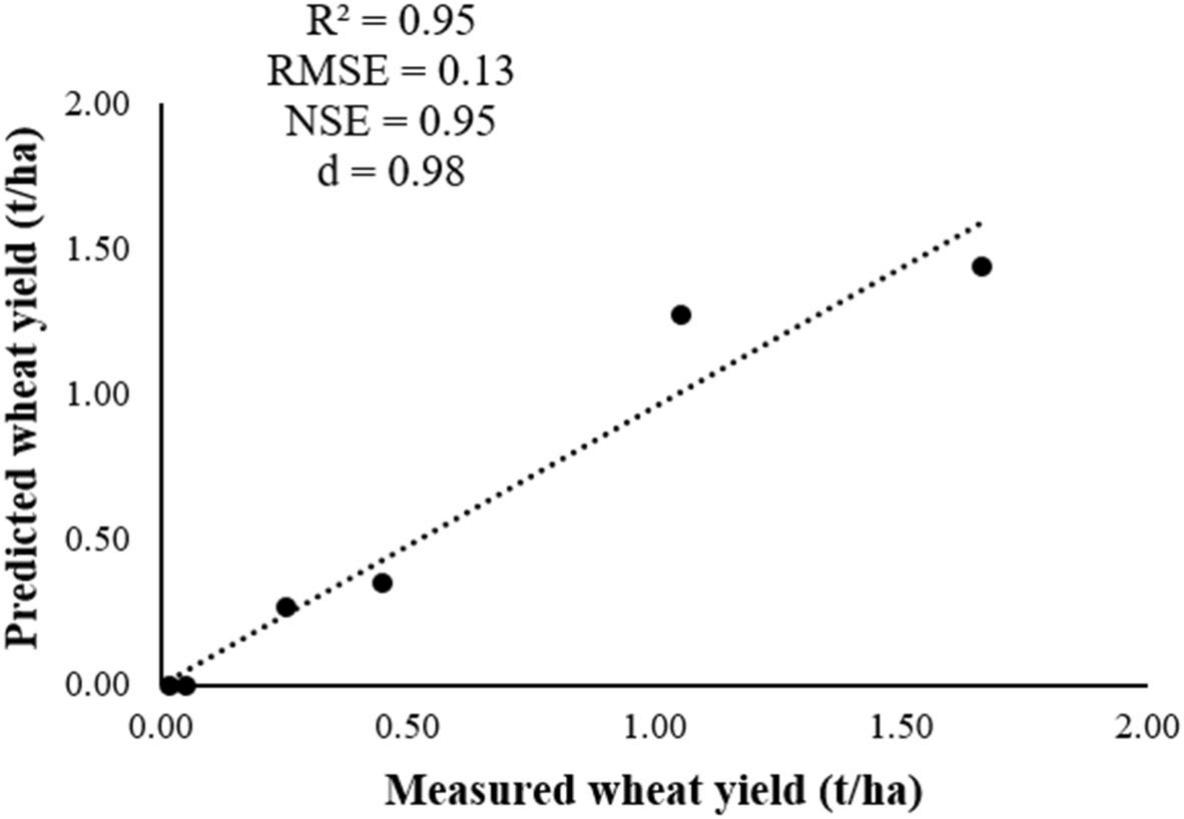
Predicted versus measured wheat yield of Marzak cultivar.

To assess whether the model provided the right answer (wheat yield) for the right reasons, we compared the wheat yield measurements for 6 years to APSIM simulations output (Table 2; Fig. 16). Three parameters are found to be the most sensitive with the relative sensitivity values such as: photoperiod, vernalization and the initial fraction of plant available water capacity (PAWC). We found that after determining these three coefficients, the model predicted well the wheat yield in the Rhamna region—Central Morocco.

**Table 2:** Statistical indices of assessing the performance of APSIM-Wheat model in predicting grain yield.

Cropping year	Grain yield (t/ha)	
	Measured	Simulated
2013–2014	1.66	1.44
2014–2015	0.26	0.27
2015–2016	0.05	0.00
2016–2017	1.06	1.27
2017–2018	0.45	0.35
2018–2019	0.02	0.00
Index		
R^2^	0.95	
RMSE	0.13	
NSE	0.95	
d	0.98

According to the performance criteria, the simulated data of wheat yield by APSIM model in comparison with the observed data allowed us to obtain a good model performance with very satisfactory values of RMSE, NSE and d of the order of 0.13, 0.95 and 0.98, respectively (Table 2; Fig. 16).

Therefore, the aforementioned indexes imply the robustness of the model in predicting wheat yield. They demonstrated that the APSIM-wheat model provided an excellent simulation performance during the determination of winter wheat yield for the Rhamna region. This indicates that the model can reflect the reality and provides a better estimation of the studied process.

### Future scenarios for wheat yield

**Figure 17:**
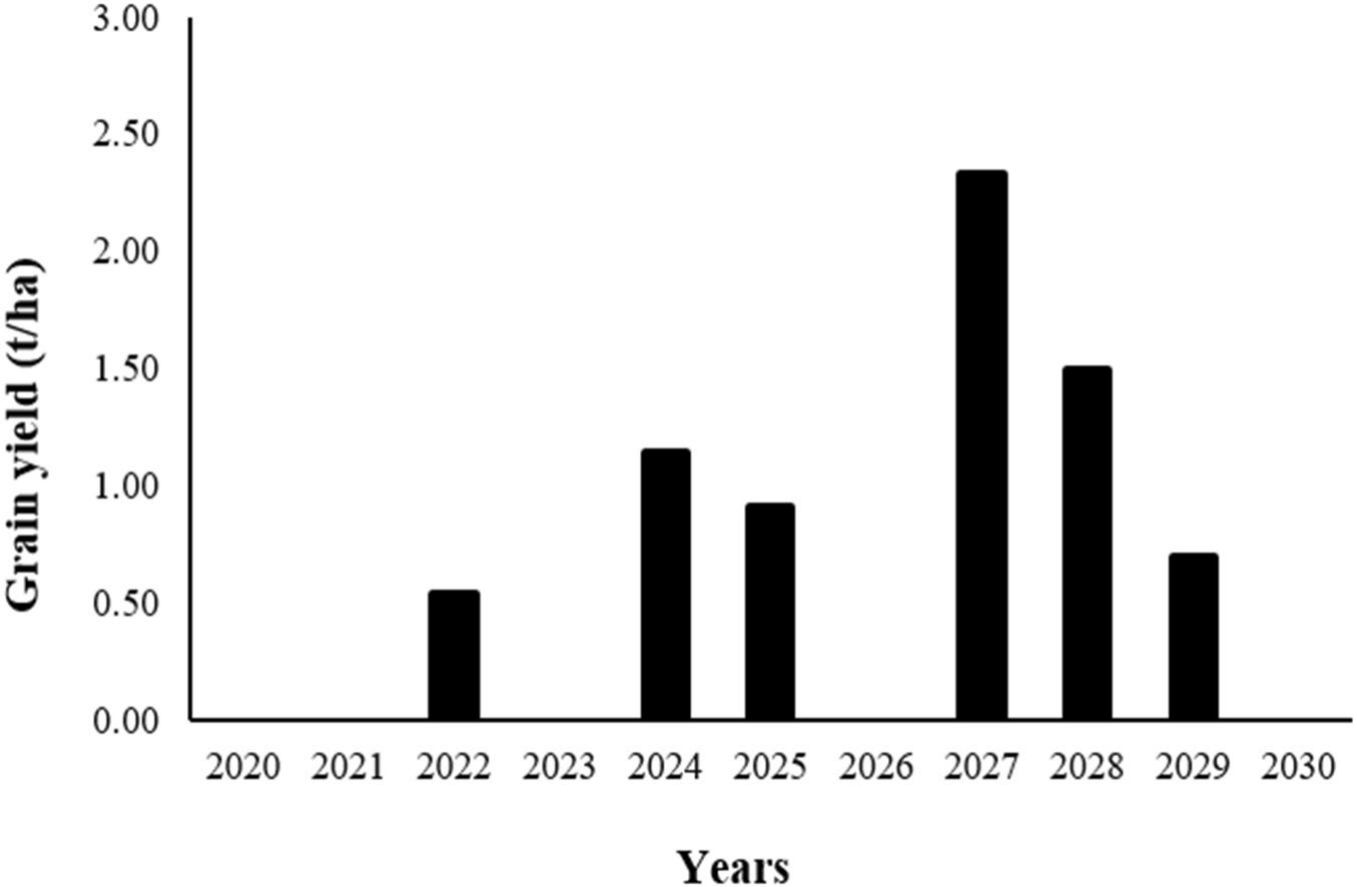
Future scenarios for wheat yield in the Rhamna region.

Various simulations were achieved using the calibrated APSIM model to estimate the wheat yield of Marzak cultivar for the future scenarios in the Rhamna region. The results show that the yield varies between 0 and 2.33 t/ha throughout the study period (Fig. 17). The maximum yield was observed for the 2026–2027 season, and the minimum yield was observed for five seasons that are: 2019–2020, 2020–2021, 2022–2023, 2025–2026, 2029–2030. The two seasons, 2023–2024 and 2027–2028, show yields between 1 and 2 t/ha (1.14 t/ha and 1.50 t/ha, respectively). The yields for the three last seasons (2021–2022, 2024–2025 and 2028–2029) are between 0 and 1 t/ha (0.54 t/ha, 0.91 t/ha and 0.70 t/ha, respectively).

The seasons that present a zero or minimal yield are consistent with the seasons in which the rainfall is limited or insufficient. In general, the yield still not satisfactory since the area is characterized by a semi-arid climate.

### Optimum sowing date of wheat

In the Rhamna region, there is no standard planting date, each farmer plants wheat crop on a date that suits him. Some of them are sown early, others during the medium or late period.

In this context, different simulation was carried out by the calibrated APSIM model to determine the optimum sowing date in the Rhamna region for the wheat crop, which was strongly affected by weather conditions and sowing date.

**Figure 18:**
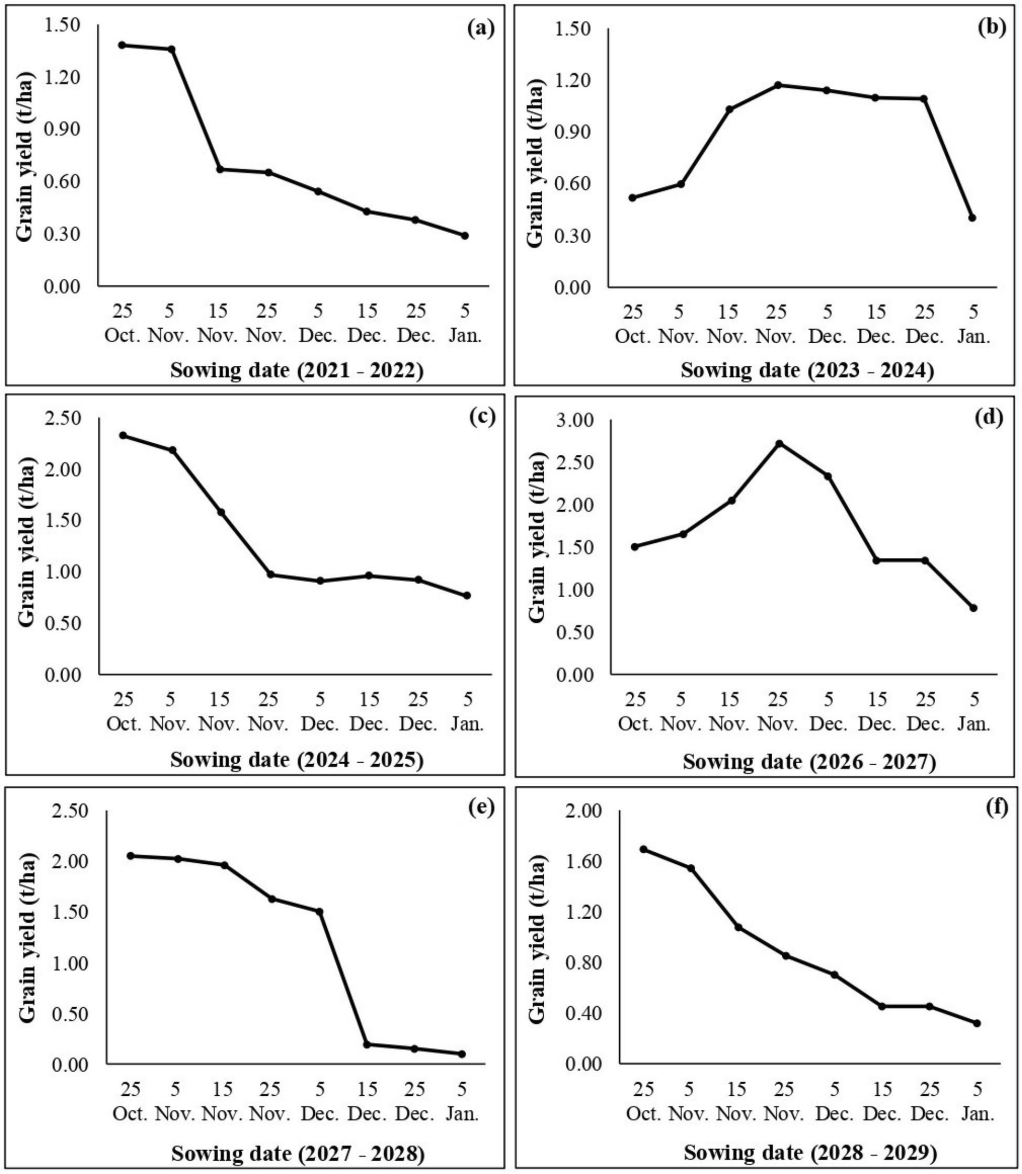
Predicted yield for different sowing dates of Marzak cultivar for the future scenarios.

Figure 18 shows that the highest yield was simulated during the early and medium sowing date of agricultural seasons, which are 25 October and 25 November, respectively. The highest yields during early sowing date were observed in four seasons, which are: 2021–2022, 2024–2025, 2027–2028 and 2028–2029. Besides, the highest yields during medium sowing date were spotted in two seasons: 2023–2024 and 2026–2027.

The agricultural season of 2021–2022 ranged from 0.29 to 1.38 t/ha, the highest and lowest yields were simulated for 25 October and 5 January sowing dates, respectively. Delaying the sowing date from 25 October to 5 January decreased yield of 1.09 t/ha. Further, the maximum value brought about in an increase in yield of 0.84 t/ha in comparison with the yield of the same season simulated by APSIM model.

Regarding the agricultural season of 2024–2025, the yields varied from 0.76 to 2.32 t/ha which were predicted for 5 January and 25 October, respectively. The yield decreased by approximately 1.56 t/ha, with a delay in the sowing date from 25 October to 5 January. In addition, the maximum value brought about an increase in yield of about 1.41 t/ha in comparison with the yield of the same year.

Concerning the year of 2027–2028, the yield ranged from 0.10 to 2.05 t/ha. The lowest yield was noticed through sowing on 5 January, and the highest yield through sowing on 25 October. Delay in sowing date from 25 October to 5 January has resulted in a decrease in yield with about 1.95 t/ha. Besides, the maximum yield obtained showed an increase in comparison with the yield of the same year with a value of 0.55 t/ha.

Relating to the average predicted yield for the year of 2028–2029, it varied from 0.32 to 1.69 t/ha. Minimum and maximum yields were simulated for 5 January and 25 October sowing dates, respectively. Delay in sowing date from 25 October to 5 January resulted in a yield diminution of about 1.37 t/ha. Furthermore, the maximum yield obtained showed an increase in comparison with the yield of the same season with a value of 0.99 t/ha.

Otherwise, the agricultural season of 2023–2024 ranged from 0.40 to 1.17 t/ha, the highest and lowest yields were simulated for 25 November and 5 January sowing dates, respectively. Delaying the sowing date from 25 November to 5 January decreased yield of 0.77 t/ha. In addition, the maximum value brought about an increase in yield of 0.03 t/ha in comparison with the yield of the same season simulated by the APSIM model.

Moreover, the agricultural season of 2026–2027 varied from 0.78 to 2.72 t/ha which were predicted for 5 January and 25 November, respectively. The yield decreased by approximately 1.94 t/ha, with a delay in the sowing date from 25 November to 5 January. Also, the maximum value resulted in an increase in yield of about 0.39 t/ha in comparison with the yield of the same season.

On the other hand, the four seasons that are 2020–2021, 2022–2023, 2025–2026 and 2029–2030 do not give significant results because they corresponded to years of drought, especially during the growing period, which led to multiple impacts on wheat yield.

### Wheat varieties

After the calibration of the APSIM model, different scenarios were carried out under the same conditions by comparing the yield of other varieties with the yield of the Marzak cultivar used in the Rhamna region.

**Figure 19:**
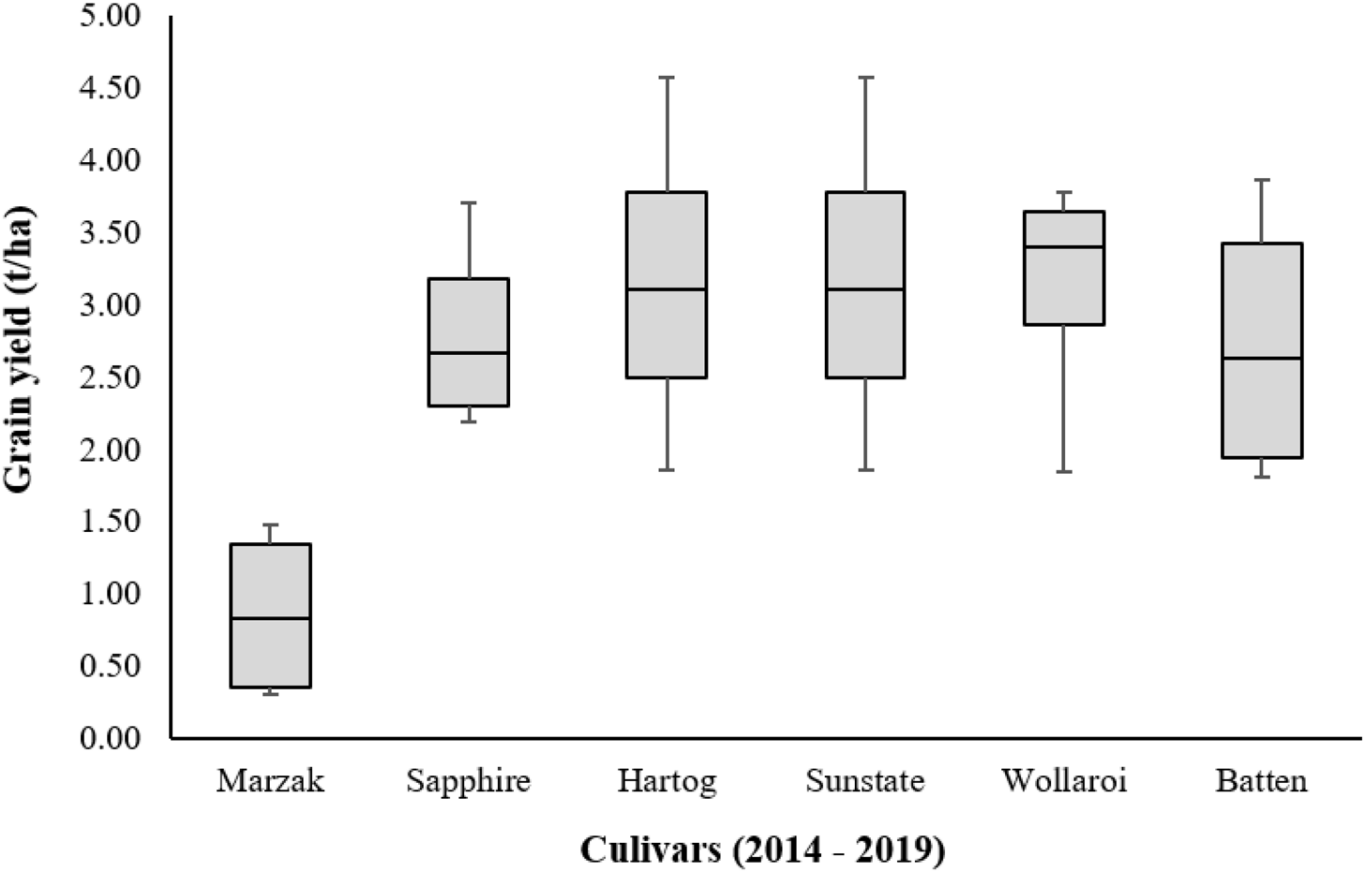
Comparison of several varieties simulated at the same conditions of the study area for the calibrated period (2014–2019).

**Figure 20:**
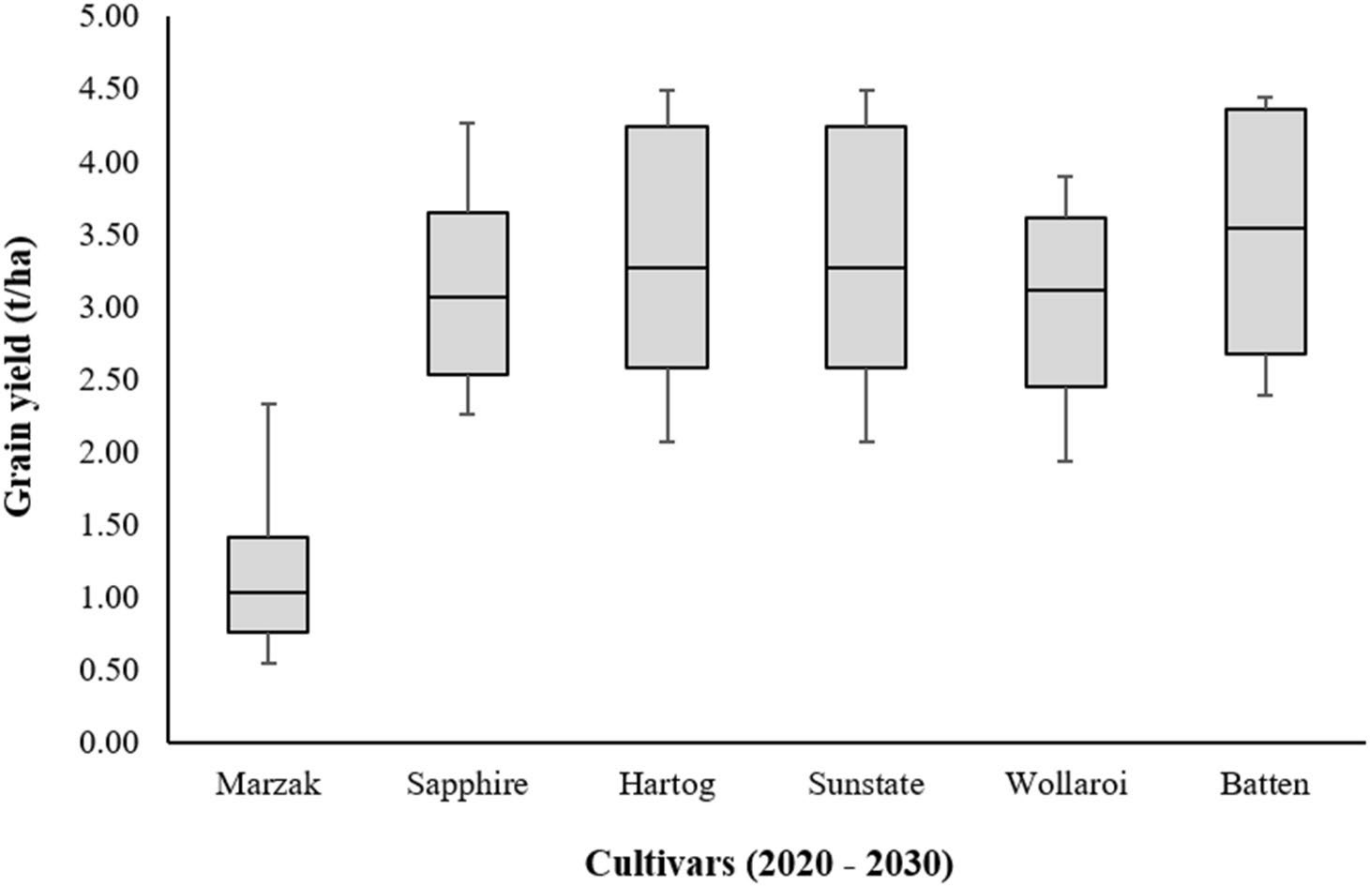
Comparison of several varieties simulated at the same conditions of the study area for the future scenarios (2020–2030).

The varieties which exhibited maximum values compared to the Marzak variety are presented in Fig. 19 during the calibration period, and in Fig. 20 during the future scenarios period.

The maximum value resulted from scenarios of cultivars for the calibration period (Fig. 19) was observed on two varieties of wheat, which are Hartog and Sunstate. The minimum and maximum value for the two cultivars varied from 1.86 to 4.57 t/ha, respectively. The highest and lowest yield was increased by values of 3.13 t/ha and 1.55 t/ha compared to Marzak cultivar, respectively. Also, the Batten variety ranged from 1.81 to 3.86 t/ha. These values show an increase of 1.5 and 2.42 t/ha, respectively. Regarding Wollaroi, it varied between 1.84 and 3.78 t/ha, which shows an increase of 1.53 and 2.34 t/ha, respectively. Moreover, Sapphire reaches maximum values relative to the crop used in the region which ranged between 2.19 and 3.71 t/ha. These values show an increase of 1.88 and 2.27 t/ha, respectively.

Similarly, as calibration period, Hartog and Sunstate cultivars show also maximum value during the future scenarios period (Fig. 20). The minimum and maximum value for the two cultivars varied from 2.07 to 4.49 t/ha, respectively. The highest and lowest yield was increased by values of 2.16 t/ha and 1.53 t/ha compared to Marzak cultivar, respectively. As regards the Batten variety, it ranged from 2.39 to 4.44 t/ha. These values show an increase of 1.85 and 2.11 t/ha, respectively. Furthermore, Sapphire cultivar varied between 2.25 and 4.26 t/ha. These values show an increase of 1.71 and 1.93 t/ha, respectively. Furthermore, Wollaroi variety ranged between 1.93 and 3.90 t/ha, which shows an increase of 1.39 and 1.57 t/ha, respectively.

These results showed us that the use of one of these varieties in the Rhamna region instead of Marzak cultivar could lead to a very satisfactory increase in wheat yield.

## Discussion

Our results showed that APSIM-Wheat model can be used as a suitable tool to investigate farm productivity thru building various scenarios management options for optimizing wheat yield under water limited environment. The ability of APSIM-Wheat model to predict yield in the semi-arid environment was confirmed by previous studies^91–95^. Application of APSIM-Wheat for grain yield simulation showed reasonable predictive results not only in semi-arid climate^43,46,96–99^, but also in arid^44,45,100^ and Mediterranean^41,42,101–103^ environments.

Determining future projections of climate data^71–76^ and integrating them into the APSIM model allowed us to get future scenarios of wheat yield in the study area. However, low yield was obtained comparing it with previous years due to either insufficient or absence of rainfall. The production of cereals in the semi-arid areas of Morocco, such as in other regions with the same climatic conditions, is strongly affected by inadequate or poorly distributed rainfall^91,104^. The upsurge of minimum/maximum temperature has been noticed during the recent decades^105^. The rise of temperature in the future years will lead to global warming thereby exasperating the drought incidence and affecting the precipitation pattern. This trend of climate variability justifies that the damage to agriculture and water scarcity has already been started and will be proliferated further over the next few years. Similar projections were reported on future climate which carried out using various climate models including multilevel mixed-effects model in the Moroccan context^74,106–111^, as well as Northern Africa^112–114^.

For this reason, various simulations were done to determine the optimum sowing date for the moisture deific area of Morocco. Accordingly, the simulated result confirmed that the yield obtained from plots seeded between 25 October and 25 November was higher than that sown until 05 January. The relationship between grain yield and different sowing dates of wheat are linear. The highest Predicted grain yield was obtained when it is sown in early sowing (i.e., late October) or medium sowing (i.e., late November), and decreased thereafter. The lowest grain yield was obtained in late sowing (January). This coincides with the findings of researches which affirmed that both early and medium sowing were beneficial for obtaining a highest yield, but the early sowing was more favorable to increase the wheat yield^21–26^. In addition, the results of predictions of the effect of sowing date on wheat yield using APSIM model were also consistent with many of the findings of simulations using other crop models in the semi-arid environment^34,115–118^. Therefore, extended life duration and favorable temperature especially at the grain filling stage, might be the main reason for the higher grain yield in early sowing dates^43,119,120^. While, delaying the sowing date beyond the optimum sowing date led to reduced grain yield, due to a shorter vegetative growth period of wheat^115,121^. Thus, sowing at the appropriate time can enhance the effective accumulative temperature, prolong the effective growth period of wheat, and then increase the yield^122–124^.

On the other hand, other simulations were performed using the calibrated APSIM model to test other types of cultivars, and then analyze and compare their yields with the yield of Marzak cultivars under the same conditions. From several varieties, Hartog, Sunstate, Wollaroi, Batten and Sapphire presented the highest yields in comparison with the Marzak variety. These significant results were observed for both periods, the calibration period and the future scenarios period. However, Sunstate and Hartog are the most cultivars adopted in arid and semi-arid regions, and widely used in these environments for many reasons: (i) very adequate for dry growing season, (ii) slow variety with excellent yield potential, (iii) did not suffer grain yield or grain quality losses, (iv) and possess a large resistance to herbicides and root rot^125–130^. Moreover, Batten and Sapphire are among the best performing varieties and among the earlier sown crops with highest yielding in arid and semi-arid climate^131,132^ but not suitable for the humid and sub-humid regions^133–135^. Furthermore, Wollaroi cultivar also performs well in drier areas, reputed for its high grain quality, tolerance to weathering, and resistance to crown rot and stripe rust^136–140^. This indicates that these cultivars are suitable and appropriate for our situation, and the import and the seed of them increase and enhance the wheat yield in the Rhamna region.

## Conclusion

Famers in Morocco continue growing wheat although its productivity is challenged by low and erratic precipitation; hot temperature; poor soil fertility and others. Seemingly, these production factors will aggravate in the years to come due to climate change. This study concluded that the use of APSIM model can help in evaluating the suitability of Central Morocco for wheat production. Based on this model, suitable time window for sowing and promising wheat varieties were identified that can be grown well in water deficit areas. This study recommends undertaking additional research taking into account varieties, sowing date, supplemental irrigation and nutrient and best management practices under water stressed environment. Yet, cultivar, sowing time and climate are found to be the critical factors for wheat growth hence they should be well managed.
